# Diversified Chromosome Rearrangements Detected in a Wheat‒*Dasypyrum breviaristatum* Substitution Line Induced by Gamma-Ray Irradiation

**DOI:** 10.3390/plants8060175

**Published:** 2019-06-14

**Authors:** Hongjin Wang, Zhihui Yu, Guangrong Li, Zujun Yang

**Affiliations:** Center for Informational Biology, School of Life Science and Technology, University of Electronic Science and Technology of China, Chengdu 611731, China; 201711090117@std.uestc.edu.cn (H.W.); 201621090111@std.uestc.edu.cn (Z.Y.); ligr28@uestc.edu.cn (G.L.)

**Keywords:** chromosome rearrangement, fluorescence *in situ* hybridization, gamma ray radiation, wheat‒*Dasypyrum* substitution

## Abstract

To determine the composition of chromosome aberrations in a wheat‒*Dasypyrum breviaristatum* substitution line with seeds treated by a dose of gamma-rays (200 Gy), sequential non-denaturing fluorescence *in situ* hybridization (ND-FISH) with multiple oligonucleotide probes was used to screen individual plants of the mutagenized progenies. We identified 122 types of chromosome rearrangements, including centromeric, telomeric, and intercalary chromosome translocations from a total of 772 M1 and 872 M2 plants. The frequency of reciprocal translocations between B- and D-chromosomes was higher than that between A- and D-chromosomes. Eight translocations between *D. breviaristatum* and wheat chromosomes were also detected. The 13 stable plants with multiple chromosome translocations displayed novel agronomic traits. The newly developed materials will enhance wheat breeding programs through wheat‒*Dasypyrum* introgression and also facilitate future studies on the genetic and epigenetic effects of translocations in wheat genomics.

## 1. Introduction

Radiation mutagenesis technology has been widely used to generate novel mutants for expanding breeding resources. Since E. R. Sears successfully transferred a leaf rust resistance gene *Lr9* from *Aegilops umbellulata* Zhuk. to wheat by ionizing radiation [1], this technology has been effectively applied to wheat and related species for developing interspecific translocations [2,3,4,5]. Several studies have revealed that the chromosomal rearrangements induced by ^60^Co-γ rays in the amphiploid reduced fertility and genomic stability in subsequent generations [6,7,8]. Wheat‒alien disomic addition and substitution lines are usually considered to be candidate materials for inducing chromosomal translocations through radiation treatment. A number of translocations have been obtained successfully by irradiation on self-pollinated seeds, spikes, and pollen at meiotic stages from the intergeneric lines between wheat and more distant species such as *Leymus*, *Agropyron*, *Secale*, *Thinopyrum*, and *Dasypyrum* [5,9,10,11,12,13,14]. Wheat‒alien chromosome translocation lines have also been irradiated with gamma rays for producing novel genetic variability [15,16,17]. As a result, many radiation-induced genetic resources shared between wheat and related species have been developed for breeding projects.

As an important relative of wheat, *Dasypyrum breviaristatum* (V^b^V^b^, 2*n* = 2x = 14 & VVV^b^V^b^, 2*n* = 4x = 28) is different from the annual diploid *Dasypyrum villosum* (VV, 2*n* = 2x = 14) in genome composition [18]. *D. breviaristatum* possesses agronomically valuable genes, such as resistance to biotic stress, high protein quality, and drought adaptation, and represents a potentially promising resource for wheat improvement globally [19]. In the past few decades, great progress has been made in wheat‒*D. villosum* hybridization and some beneficial resistance genes originating from *D. villosum* have been successfully transferred into the common wheat background, such as *Pm21*, *Pm55*, and *Pm62*, for powdery mildew resistance [17,20,21], and *Sr52* for stem rust resistance [22]. The study of *D. breviaristatum* is relatively slow due to the difficulty of hybridization between *Triticum* and *D. breviaristatum* [23]. A fertility-improved partial amphiploid TDH-2 (AABBV^b^V^b^), was produced and identified from progeny of a Chinese Spring wheat‒*D. breviaristatum* amphiploid following self-pollination [24]. One wheat‒*D. breviaristatum* 7V^b^ disomic addition line and a 2V^b^(2D) substitution line with high resistance to wheat stripe rust races, and a 1V^b^ substitution line with novel HMW-GS gene have been characterized by integrated molecular and concise cytogenetic techniques sequentially [25,26,27].

The non-denaturing fluorescence *in situ* hybridization technique (ND-FISH), a convenient and economical means for identifying chromosomes, has been utilized for characterizing a large number of materials in a short time [28]. Both simple sequence repeats (SSRs) and non-SSR oligonucleotide (oligo) probes have been developed for ND-FISH assays to target the species-specific chromosomes or segments [29,30,31]. Furthermore, synthesized tandem repeat-based oligos are equally efficient for the identification of wheat and alien chromosomes [32]. Moreover, oligo probes including Oligo-B and Oligo-D for distinguishing wheat B- and D-genomes [33], Oligo-pDb12H for targeting *Dasypyrum* chromosomes have also been developed [34]. These oligo probes have allowed researchers to easily identify some specific chromosomal rearrangements and reciprocal translocations involving the three sub-genomes of wheat and *Dasypyrum* genome through ND-FISH analysis.

In the present study, we report extensive chromosomal rearrangements in the *D. breviaristatum* 2V^b^(2D) [25] and 2V^b^-1(2D) [27] substitution lines induced by gamma ray radiation. The breakage and re-fusion among wheat chromosomes and between wheat and *D. breviaristatum* chromosomes were investigated by using ND-FISH. The potential usefulness of these chromosome structural variations for wheat quality improvement is also discussed.

## 2. Results

### 2.1. ND-FISH Karyotype of 2V^b^(2D) Substitution Lines

**Figure 1:**
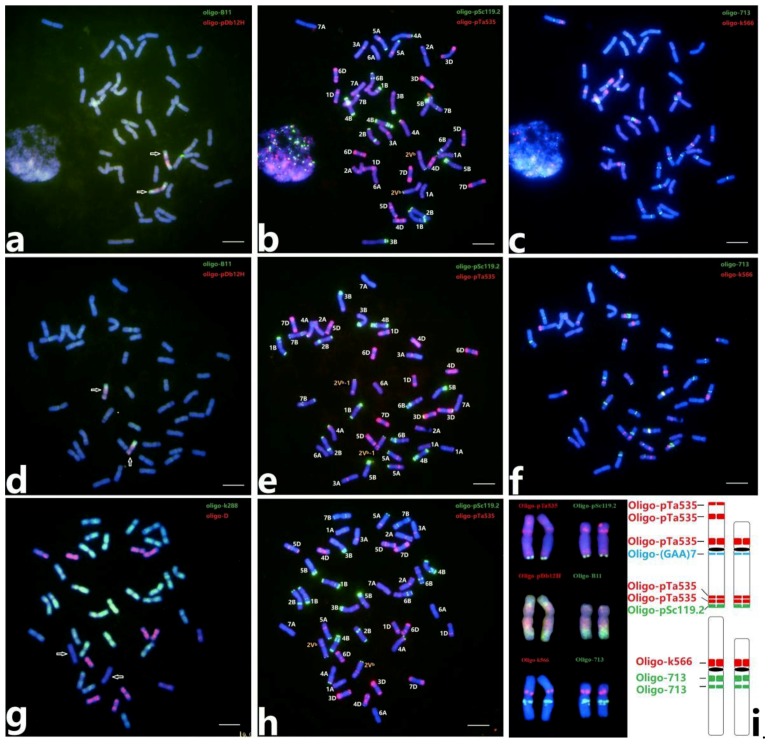
The sequential ND-FISH patterns of wheat‒*D. breviaristatum* substitution lines D11-5 (2V^b^/2D) and D2230 (2V^b^-1/2D) with multiple probes. The probes Oligo-B11 (green) + Oligo-pDb12H (red) (**a**,**d**), Oligo-pSc119.2 (green) + Oligo-pTa535 (red) (**b**,**e**,**h**), Oligo-k566 (red) + Oligo-713 (green) (**c**,**f**), Oligo-k288 (green) + Oligo-D (red) (g) were presented, respectively. Arrows indicated the *D. breviaristatum* chromosomes. The karyotypes of *D. breviaristatum* chromosomes 2V^b^ and 2V^b^-1 are shown (**i**). Bars represent 10 μm.

Wang et al. [27] identified the two wheat‒*D. breviaristatum* 2V^b^ introgression lines D11-5 (2V^b^) and D2230 (deletion 2V^b^-1) by Oligo-pSc119.2, Oligo-pTa535 and Oligo-(CAA)_7_, and constructed a karyotype of chromosome 2V^b^. In the present study, sequential ND-FISH was performed with new Oligo probes Oligo-pDb12H, Oligo-B11, Oligo-k288, Oligo-D, Oligo-713, and Oligo-k566 for distinguishing the wheat and 2V^b^ chromosomes, and then we obtained an updated FISH karyotype for 2V^b^ (Figure 1). The *D. breviaristatum* chromosome 2V^b^ can be clearly detected by probes Oligo-pDb12H and Oligo-B11, the former with distinct signals in the pericentromeric region of 2V^b^, and the latter with signals on the telomeric and sub-telomeric regions in both arms of of 2V^b^ (Figure 1a). Compared to chromosome 2V^b^, the chromosome 2V^b^-1 showed a deletion without Oligo-B11 signals in the short arm (Figure 1d). The ND-FISH with the probe Oligo-k566 revealed strong signals in the pericentromeric region of the short arm of 2V^b^, and the probe Oligo-713 produced two different intensity signals in the pericentromeric region of the long arm of 2V^b^ (Figure 1c). Based on the FISH patterns of five probes, including Oligo-pSc119.2, Oligo-pTa535, Oligo-(CAA)_7_, Oligo-k566 and Oligo-713, an updated map of 2V^b^ was constructed as shown in Figure 1i. The physical region of 2V^b^L was classified to seven regions. Additionally, a new Oligo-k288 could replace the previous pTa-k288 and produce diffuse signals enriched in both A- and B-genome chromosomes, while two chromosomes 2V^b^ showed no signals (Figure 1g). The combination of the two probes Oligo-k288 and Oligo-D can be applied for the analysis of the translocations generated between the AB-genomes and the D-genome.

### 2.2. Chromosome Variations in M0 Seeds

**Figure 2:**
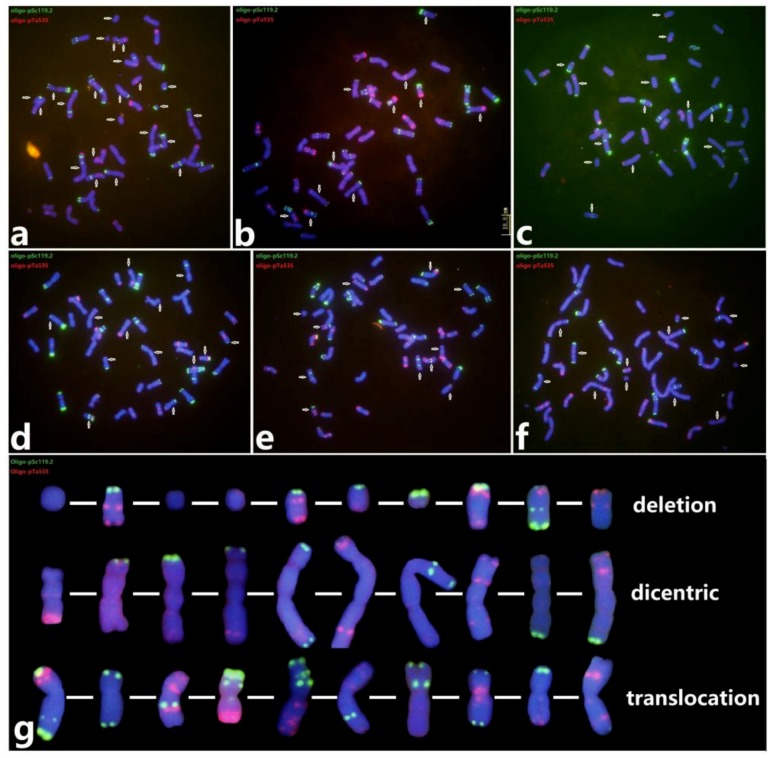
ND-FISH detection of chromosomal structure aberrations in M0 generation with Oligo-pSc119.2 (green) and Oligo-pTa535 (red). Six representative plants, 16-W289 (**a**), 16-W320 (**b**), 16-W695 (**c**), 16-W462 (**d**), 16-W777 (**e**), and 16-W473 (**f**), were selected for displaying huge structural aberrations in root tip metaphase chromosomes. The arrows indicate abnormal chromosomes. The karyotypes of deleted, dicentric, and translocated chromosomes are shown (**g**).

A total of 268 D11-5 and 179 D2230 seeds were irradiated with a dosage of 200 Gy (1.00 Gy/min). About 34.3% of D11-5 and 51.4% of D2230 seeds germinated and produced selfed progenies. For the treatment of a dosage of 200 Gy (1.86 Gy/min), a total of 343 seeds were investigated, and about 24 (12.0%) seeds of D11-5 and 15 (10.5%) seeds of D2230 gave rise to offspring. As expected, only a relatively small proportions of seeds could produce progeny after γ-irradiation, as expected. In total, 109 M0 seeds of D11-5 and D2230 were randomly selected and their mitotic metaphase chromosomes in root tips were analyzed by ND-FISH with the probes Oligo-pTa535 and Oligo-pSc119.2. About 102 seeds (93.6%) contained variations in chromosome structures, in which in each cell about five to 23 chromosomes showed changed FISH signals (Figure 2a‒e). Three types of structural changes in chromosomes, including deletions, dicentromerics, and translocations, are defined in Figure 2g. Given the huge changes to chromosomes, only 52 seedlings (47.7%) could grow into adult plants. It is possible that mutations have occurred in somatic chromosomes that may be able to recover to some extent.

### 2.3. The Wheat‒Wheat Translocations in M1 and M2

A total of 772 M1 and 872 M2 plants for karyotype analysis were successfully screened by ND-FISH using probes Oligo-pSc119.2 and Oligo-pTa535. There were 495 individuals (64.12%) of the M1 generation that displayed no discernible alterations to the ND-FISH signals. Among the remaining 277 plants, we observed four types of chromosome variation, including homozygous translocations, heterozygous translocations, unidentified translocations, and chromosomal number variation, with 58 (7.51%), 82 (10.62%), 69 (8.94%), and 68 (8.81%), respectively (Figure S1). In all, 157 chromosome translocation events were identified among wheat genomes, consisting of 90 different types including 71 Robertsonian translocations and 19 small segment translocations. In addition, a wheat‒wheat tri-homozygous translocation (T3AS.4BS; T5BS.4BL; T3AL.5BL), and a pericentric inversion (T5BS-5BL.5BS-5BL) were also found.

**Figure 3:**
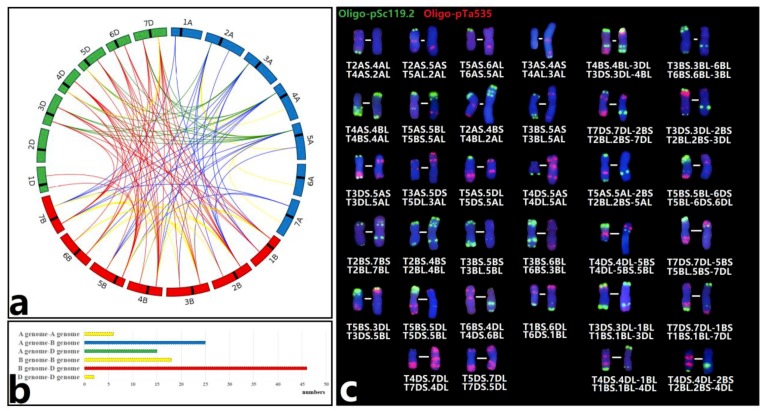
The profile of 112 interchromosomal translocation events among A-, B- and D-genome chromosomes by ND-FISH with Oligo-pSc119.2 and Oligo-pTa535 probes. (**a**) Yellow lines indicated the translocations occurred in the same genome; blue lines showed the rearrangements happened between A-genome and B-genome; the translocations occurred between B-genome and D-genome are marked with red lines; green lines show the translocations between A-genome and D-genome. (**b**) The number of interchromosomal translocation events in six different combinations was calculated. (**c**) Several chromosomal rearrangements, including 22 Robertsonian translocations and 12 small-segment translocations, are shown.

A total of 112 wheat‒wheat chromosomal rearrangements (except a tri-translocation and a inversion) in the M2 generation were observed (Figure 3a, Table S1), of which seven small-segment translocations and 15 Robertsonian translocations were identical to those from M1 plants. Different frequencies of translocation were observed in the B-genome (47.77%), D-genome (29.02%) and A-genome (23.21%). The reciprocal translocations between B- and D-chromosomes (46) was the highest, while the translocations among D-genome chromosomes (2) were the lowest (Figure 3b). The typical FISH karyotypes of 22 reciprocal Robertsonian translocations and 12 non-Robertsonian translocations are indicated in Figure 3c. Additionally, among individual wheat chromosomes, over 17 translocation events were recorded for chromosomes 5B, 2B, 7B, and 7D, while only seven translocation events involving 1A, 6A, and 7A were found.

Furthermore, other chromosome structural modifications involving small segmentation exchanges in M2 plants were also discovered by ND-FISH signal variations. For instance, a strong Oligo-pTa535 signal was added in the terminal region of 4AS (Figure S2a,d), a distinct Oligo-pSc119.2 signals appeared in the terminal regions of 2AL, 5BL, 7BS and 7DL (Figure S2b–e), and a weak Oligo-pSc119.2 signal was inserted in the intercalary regions of long arm of 3BL (Figure S2e). These modifications of FISH sites on different chromosomes may be caused by rapid amplification of repetitive DNA sequences, induced by gamma irradiation.

### 2.4. Determination of Breakage Points for Non-Robertsonian Translocation

**Figure 4:**
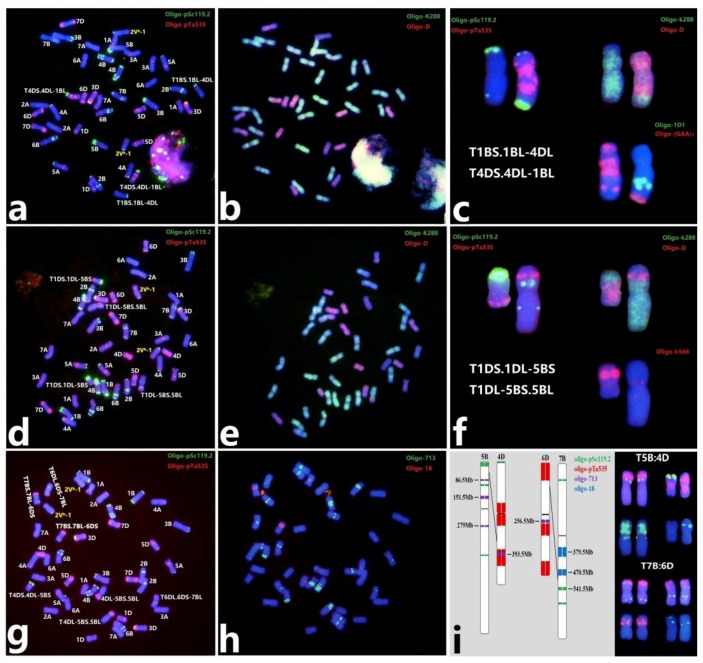
Sequential ND-FISH analysis of the metaphase chromosomes of wheat‒wheat translocation lines. T1BS.1BL-4DL and T4DS.4DL-1BL translocation (**a**,**b**) and T1DS.1DL-5BS and T1DL-5BS.5BL translocation (**d**,**e**) with the probes Oligo-k288 (green) and Oligo-D (red), Oligo-pSc119.2 (green), and Oligo-pTa535 (red); the A line included two non-Robertsonian translocation events involving 5B, 4D, 6D, and 7B, that were analyzed using Oligo-pSc119.2 (green), Oligo-pTa535 (red), Oligo-713 (green), and Oligo-18 (red) as probes (**g**,**h**). Images show enlargements of the FISH pattern of chromosomes with structural changes (**c**,**f**,**i**). Arrows and black lines indicate the breakage points.

Non-Robertsonian translocations involving two or three pairs of homologous chromosomes pairs were characterized by ND-FISH with multiple probes. As shown in Figure 4, two small segmental translocations including T1BS.1BL-4DL and T4DS.4DL-1BL, T1DS.1DL-5BS, and T1DL-5BS.5BL were identified by sequential ND-FISH using the probes Oligo-k288, Oligo-D, Oligo-pSc119.2, and Oligo-pTa535. The translocation breakpoints were the subtelomeric regions of 1BL and 4DL (Figure 4a-c), and close to the middle of chromosomes 5BS and 1DL (Figure 4e,f), respectively. Another line, 17-M743, contained two types of non-Robertsonian translocation events involving 5B, 4D, 6D, and 7B, and was characterized using Oligo-pSc119.2, Oligo-pTa535, Oligo-713, and Oligo-18. The breakpoints could be defined precisely in the predicted physical regions using the integrated map of oligo probes published by Lang et al. [35]. The breakpoint on 5B was located to the regions of 4‒86.5Mb, and the breakpoint on 7B at the region of 470.5‒541.5Mb (Figure 4g–i).

In total, 26 non-Robertsonian translocations among the A-, B- and D-genome chromosomes were investigated (Figure S3). Except for 1A, 3A, 6A, and 2D, 17 wheat chromosomes were involved. The majority of breakpoints were located on B-genome chromosomes (26, 50%) and D-genome chromosomes (21, 40.38%), which were more frequent than those of the A-genome (5, 9.61%). Four chromosome arms, 2BS (6), 5BS (5), 4DL (5), and 6DS (4), accumulated the most translocation breakage points. These breakpoints were mainly distributed at the telomeric or subtelomeric region of these chromosomes.

### 2.5. Identification of Wheat‒D. breviaristatum Translocations

**Figure 5:**
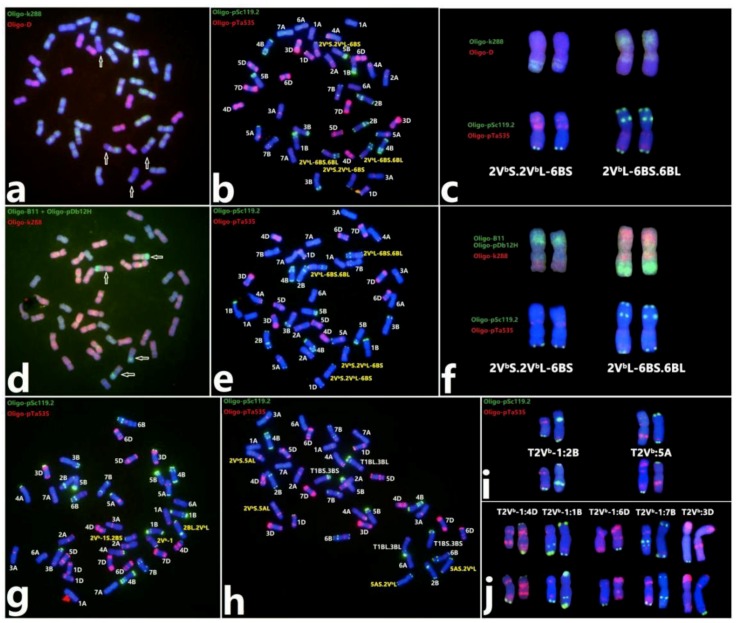
Summary of the irradiation-induced chromosome translocations that occurred between wheat chromosomes and *D. breviaristatum* chromosomes. The translocation T2V^b^:6B was confirmed using the probes Oligo-k288 (green) and Oligo-D (red) (**a**), the combination of the three probes Oligo-pDb12H (green), Oligo-B11 (green), and Oligo-k288 (red) (**d**), and Oligo-pSc119.2 (green) + Oligo-pTa535 (red) (**b**,**e**) by sequential ND-FISH analysis. Two translocation chromosomes, T2V^b^S.2V^b^L-6BS and T2V^b^L-6BS.6BL, showed the breakpoint closed to the centromere (**c**,**f**). Karyotype of wheat‒*D. breviaristatum* 2V^b^ translocation lines T2V^b^-1:2B and T2V^b^:5A (**g**,**h**). Seven types wheat‒*D. breviaristatum* 2V^b^ translocation chromosomes were compared to the corresponding normal chromosomes below them (**i,j**). White arrows indicate the translocation chromosomes T2V^b^:6B.

A total of eight wheat‒*D. breviaristatum* translocations were characterized by ND-FISH with reference to the standard FISH idiogram of wheat and 2V^b^ chromosome [25,32]. Six lines were completely fertile to produce stable generations and were used for determination of the breakpoints by FISH by the combination of probes Oligo-k288, Oligo-D, Oligo-B11, and Oligo-pDb12H. The translocations of T2V^b^S.2V^b^L-6BS and T2V^b^L-6BS.6BL apparently involved breakage points close to the centromere of 2V^b^L and the satellite of 6BS (Figure 5a–f). Four lines, 17-S898 (T2V^b^:3D), 17-M560 (T2V^b^-1:6D), 17-M325 (T2V^b^-1:7B), and 18-M939 (T2V^b^:5A), were determined to be non-Robertsonian translocations (Figure 5h–j), and line 17-S497 has a homologous Robertsonian translocation of T2V^b^-1:2B (Figure 5g,i) (the representation of translocations according to Badaeva et al.) [36]. The lines 17-S295 and 17-S300 carrying T2V^b^-1:1B and T2V^b^-1:4D chromosomes, respectively (Figure 5j), were sterile, not producing seeds for subsequent progeny. The results indicated that the proximal region of 2V^b^L (83.33%) generated more breakage points than that of the centromere of 2V^b^ (16.67%). In addition, we found four structural variations involving 2V^b^ and 2V^b^-1: an Oligo-pSc119.2 signal was added at the end of 2V^b^S (Figure S4a), a pericentric inversion occurred in the proximal region of 2V^b^ (Figure S4b,e), and the other two inversions were characterized in the distal of 2V^b^ and 2V^b^-1, respectively (Figure S4c–e).

### 2.6. Survey of Agronomic Traits

Seven important agronomic traits of 35 M1 plants with chromosome translocations and an equal number of normal M1 plants, including D11-5 (13) and D2230 (22), respectively, were measured under field conditions during 2016‒2017 (Table 1). Except for spikelet number, the other six traits showed higher variability for the plants with chromosome translocations compared to the control plants. A large range of genetic variations was observed in 1000-grain weight, plant height, and tiller number, and the coefficient of variation (CV) of these was greater than 15%. In addition, the range of 1000-grain weight of the plants with chromosome translocations were significantly increased compared to the plants without translocations. A translocation line 17-M819 (T5AS.5BL and T5BS.5AL) showed excellent performance in the seeds, with the highest 1000-kernel weight (69.33 g) and the longest 10-kernel length (9.42 cm). One line 17-M776 (T3BS.4DL and T4DS.3BL) showed the maximal tiller number at 18, and a lower plant height of 61 cm. These results indicated that some M1 plants with chromosome translocations possessing favorable new traits might be selected for the improvement of wheat.

## 3. Discussion

The ND-FISH assay with short synthetic oligo probes provides a low-cost, high-throughput way to identify the karyotype variation of wheat more rapidly than with the conventional method [28,31,32,37,38]. Recently developed oligo probes such as Oligo-1162 [30], Oligo-Ku [39], Oligo-B and Oligo-D [33], Oligo-pDb12H [34], and Oligo-B11 [40] have been applied to ND-FISH in order to distinguish the different genomes. In the present study, a new oligo probe, named Oligo-k288, was designed according to the LTR sequence of pTa-k288 [41], which showed full hybridization of the entire chromosomes of the A- and B-genomes simultaneously. Combined with the probe Oligo-D, Oligo-k288 can be used to detect alien chromosomes in the wheat background and easily identify the translocations occurring between A-, B-, and D-genome chromosomes (Figure 4b,e). Furthermore, our results indicated that two probes, Oligo-pDb12H and Oligo-B11, are suitable for characterizing the V^b^-genome of *D. breviaristatum* chromosomes, and their diffuse signals are similar to chromosomes of the J^S^ genome [34]. In addition, over 100 translocation events were identified by ND-FISH using the probes Oligo-pSc119.2 and Oligo-pTa535, which were complemented by the probes Oligo-713, Oligo-k566, Oligo-1D1, and Oligo-18 [35]. For the two translocation events of 5B and 7B (Figure 4i), the breakpoints were physically located in 4‒86.5Mb of 5B and 470.5‒541.5Mb of 7B, respectively. The results suggested that the ND-FISH with multiple probes can facilitate the precise identification of translocations related to specific chromosomal regions.

A high frequency of chromosomal rearrangements in plants can be induced by radiation [3,7,42,43]. We analyzed 112 chromosome translocations among wheat genomes and eight wheat‒*D. breviaristatum* translocations obtained from D11-5 and D2230 induced by ^60^Co γ irradiation in the M1‒M2 generations. The translocation events between the B-genome and the D-genome reached 41.07% of total translocations, which was similar to the report by Zhang et al. [44], since B-genome chromosomes contained a large content of heterochromatin [45,46]. Above all, centromeric translocations were induced at a large proportion (76.79%) in this study, which may have resulted from misdivision and re-fusion among univalents during meiosis of mutated plants [47,48]. Although the molecular mechanisms of non-centromeric translocations have still not been explained clearly, the breakpoints are usually enriched in the heterochromatin region [36,49]. Our results indicated that the breakpoints of 26 small segmental translocations were mainly distributed in the telomeric and subtermeric regions of the B- and D-genome chromosomes (Figure S3). It is likely that the telomeric sequences of B- and D-genome chromosomes enriched the repeats of pSc119.2 (120-bp family sequences) and pTa535 (*Afa* family sequence), respectively [41,50,51,52]. Fewer breakpoints were observed within the A genome, which may be due to the lack of labeling probes for ND-FISH screening or, alternatively, the A-genome chromosomes being less sensitive to irradiation. Given that mutagenetic chromosome rearrangements occurred in the repetition-rich regions, more probes need to be developed for further exploring the breakpoint positions of different chromosomes.

Chromosome translocations are widespread in the wheat genomes, which may lead to the alteration of gene expression, and affect the formation of various agronomic traits [53]. A number of wheat varieties possessed translocated chromosomes, including T5BS.7BS, T1BL.1RS, and T6AL.6VS, which were developed and released due to high levels of resistance and other desirable traits [54,55,56]. Also, multiple chromosome translocations can not only improve disease resistance but also broaden the genetic basis for the local adaptation of wheat varieties [57,58]. In this study, these stable translocation lines showed a large range of genetic variation in agronomic traits. Several translocation lines demonstrated remarkable superiority in one or two traits, which will have potential value for improving wheat traits. These results indicated that novel agronomic traits for facilitating wheat breeding programs can be obtained by inducing irradiation through inheritable multiple chromosome translocations. Furthermore, a wheat‒*D. breviaristatum* homologous translocation line, T2B:2V^b^-1, can serve as a bridge material for transferring the stripe rust resistance gene(s) located on 2V^b^L (FL 0.40–1.00) to the wheat background [27]. These non-Robertsonian translocations have high potential to clarify our understanding of the contribution of chromosomal rearrangements to agronomic traits in the wheat genome.

## 4. Materials and Methods

### 4.1. Plant Materials

The homozygous wheat‒*D. breviaristatum* 2V^b^ (2D) line D11-5 and 2V^b del^(2D) line D2230 [25,27] were developed at the University of Electronic Science and Technology of China. Dry seeds of D11-5 and D2230 were irradiated using ^60^Co γ-rays at the dosages of 200 Gy with two different dose rates of 1.00 Gy/min and 1.86 Gy/min at the Institute of Biological and Nuclear Technology, Sichuan Academy of Agricultural Sciences, Chengdu, China. The individual plants from the mutagenized generation (M0) and their self-pollinated progenies (M1‒M2) were analyzed using ND-FISH.

### 4.2. Cytological Preparation

Seeds were germinated on wet filter paper in Petri dishes for 24 h at 22 °C and then kept at 4 °C for 24 h, followed by 22 °C for 24 h to synchronize cell division. Root tips were excised when they were about 2‒3 cm long, and then were treated with N_2_O at 1.0 MPa pressure for 2 h. After fixing for 10 min in ice-cold 90% acetic acid, root tips were washed twice in double-distilled water and stored in 70% ethanol at ‒20 °C [59]. Subsequently, chromosome preparations were made from the preserved root tips according to the method described by Han et al. [60].

### 4.3. DNA Probes and Labeling

The previously reported oligonucleotide probes Oligo-pSc119.2, Oligo-pTa535 [32], Oligo-k566, Oligo-713 [61], Oligo-45, Oligo-18, Oligo-D, Oligo-B [33], Oligo-pDb12H [34], Oligo-1D1 [35], Oligo-(GAA)_7_ [37], and Oligo-B11 [40] were synthesized for identifying the wheat and *D. breviaristatum* chromosomes. A new oligo probe, Oligo-k288, was labeled for replacing the long terminal repeat (LTR) probe pTa-k288 for specific hybridization of A- and B-genomes [41]. The oligo probes were synthesized by Shanghai Invitrogen Biotechnology Co. Ltd. (Shanghai, China). All of them were labelled with 6-carboxy fluorescein (6-FAM) for green or 6-carboxytetramethylrhodamine (TAMRA) for red signals, respectively.

### 4.4. ND-FISH and Sequential FISH Analysis

The ND-FISH for the prepared chromosomes was performed according to the methods described by Fu et al. [30]. Three rounds of sequential ND-FISH were performed for precise dissection of the different types of chromosome rearrangements between wheat‒wheat and wheat‒*D. breviaristatum* 2V^b^. Firstly, ND-FISH by probes Oligo-k288, Oligo-D, Oligo-B11, and Oligo-pDb12H was used to distinguish the recombination among different genomes. After capturing images, the chromosome preparations were washed gently with 70% ethanol followed by 50% formamide to remove the probes. For the second round of ND-FISH, Oligo-pSc119.2 and Oligo-pTa535 were used to identify the individual chromosomes. Finally, Oligo-713, Oligo-1D1, Oligo-(GAA)_7_, Oligo-45, and Oligo-18 were used to determine the specific chromosome regions involved in the translocated chromosomes by the third round of ND-FISH. The sequences for the above oligo probes are listed in Table 2. All images were captured by a DP-70 CCD camera attached to an Olympus BX-51 microscope (Shinjuku, Tokyo, Japan) and analyzed using Adobe Photoshop (Adobe Systems Incorporated San Jose, CA, USA).

### 4.5. Agronomic Traits

All materials were planted in the field at the Xindu Experimental Station, Chengdu, China, consecutively for three years. Seven phenotypic traits, including plant height, tiller number, spike length, spikelet number per spike, 1000-kernel weight, grain length, and grain width, were recorded at the fully mature stage.

## Figures and Tables

**Table 1:** Performance and variance of agronomic traits of 35 M1 plants with chromosome translocations.

Agronomic Traits	Plants without Translocations	Plants with Translocations
1000-grain weight (g)	39.96 (23.06–60.54)	36.13 (15.68–69.33)
10-kernel length (cm)	7.84 (7.16–8.96)	7.86 (7.00–9.42)
10-kernel width (cm)	2.82 (2.18–3.26)	2.66 (1.85–3.25)
Plant height (cm)	81.91 (60.00–103.00)	80.03 (61.00–124.00)
Tiller number	8.14 (4.00–17.00)	9.31 (4.00–18.00)
Spike length (cm)	14.29 (9.80–17.30)	14.25 (9.00–17.00)
Spikelet number	26.39 (20.00–31.00)	26.83 (24.00–30.00)

The data presented are the mean (min–max).

**Table 2:** Name, sequence, and reference of oligonucleotide probes for fluorescence *in situ* hybridization (FISH) analysis.

Name	Sequences	Reference
Oligo-pSc119.2	CCGTTTTGTGGACTATTACTCACCGCTTTGGGGTCCCATAGCTAT	[32]
Oligo-pTa535	GACGAGAACTCATCTGTTACATGGGCACTTCAATGTTTTTTAAACTTATTTGAACTCCA	[32]
Oligo-pDb12H	TCAGAATTTTTAGGATAGCAGAAGTATTCGAAATACCCAGATTGCTACAG	[34]
Oligo-B11	TCCGCTCACCTTGATGACAACATCAGGTGGAATTCCGTTCGAGGG	[40]
Oligo-713	GTCGCGGTAGCGACGACGGACGCCGAGACGAGCACGTGACACCATTCCCACCCTGTCTA	[61]
Oligo-k566	ATCCTACCGAGTGGAGAGCGACCCTCCCACTCGGGGGCTTAGCTGCAGTCCAGTACTCG	[61]
Oligo-D	TACGGGTGCCAAACGAGTGTCTGAAAGACTCCTCGAGAGGAAAATGCGAA	[33]
Oligo-B	GGTTCAGGAATAGCCTCAGGAATTGGCTCAATT	[33]
Oligo-45	CGGCCGCTCCGCGCGTCGCCATCGGTTGGTCACCTCATCACCACT	[33]
Oligo-18	GTAGCAGTAGCAGTAGTA	[33]
Oligo-(GAA)_7_	GAAGAAGAAGAAGAAGAAGAA	[37]
Oligo-1D1	CGGAGTCCGTTTTGGCTCCACAAGTAGTCAAAACGTTTGTGACGACCAGATGCT	[35]

## Supplementary Materials

The following are available online at https://www.mdpi.com/2223-7747/8/6/175/s1, Figure S1: The survey of chromosomal rearrangements among 772 plants in M1 generation; Figure S2: Other chromosome modifications were identified in M1 and M2 generations; Figure S3: Twenty-six non-Robertson translocations among A-genome, B-genome and D-genome were showed; Figure S4: Four types of structural variations involving 2V^b^ and 2V^b^-1 were exhibited; Table S1: 116 materials with translocations were showed.
